# Advantages of an alternate-day glucocorticoid treatment strategy for the treatment of IgG4-related disease: A preliminary retrospective cohort study

**DOI:** 10.1097/MD.0000000000030932

**Published:** 2022-09-30

**Authors:** Sho Fukui, Takehiro Nakai, Satoshi Kawaai, Yukihiko Ikeda, Masei Suda, Atsushi Nomura, Hiromichi Tamaki, Mitsumasa Kishimoto, Sachiko Ohde, Masato Okada

**Affiliations:** a Immuno-Rheumatology Center, St. Luke’s International Hospital, Tokyo, Japan; b Center for Clinical Epidemiology, St. Luke’s International University, Tokyo, Japan; c Department of Emergency and General Medicine, Kyorin University School of Medicine, Tokyo, Japan; d Department of Rheumatology, Suwa Chuo Hospital, Nagano, Japan; e Department of Rheumatology, JOJINKAI Ushiku Aiwa General Hospital, Ibaraki, Japan; f Department of Nephrology and Rheumatology, Kyorin University School of Medicine, Tokyo, Japan.

**Keywords:** alternate-day glucocorticoid, drug adverse events, glucocorticoid, glucocorticoid toxicity index, IgG4-related disease, immunosuppressants, infection

## Abstract

Alternate-day glucocorticoid (GC) therapy is a treatment option that can reduce GC-associated adverse events. We investigated the safety and efficacy of alternate-day GC therapy in patients with immunoglobulin G4-related disease (IgG4-RD).

Medical records of patients with IgG4-RD who were followed for at least one year at St. Luke’s International Hospital, Tokyo, Japan, from 2004 to 2020 were reviewed. Patients who fulfilled comprehensive IgG4-RD diagnostic criteria were divided into alternate-day or daily GC treatment groups based on their treatment protocol. The effect of alternate-day GC therapy on glucocorticoid toxicity index (GTI) score was evaluated using multilinear analysis with adjustments for cumulative GC doses until each assessment point and propensity scores (PS) for alternate-day GC therapy. Kaplan–Meier curves and Cox proportional hazard models were used to assess the efficacy of alternate-day GC therapy for disease control.

Among the 67 patients with IgG4-RD, patients with alternate-day (n = 13) and daily (n = 31) GC treatments were analyzed after excluding 23 ineligible patients. The median (interquartile range) age was 64 (60–70) years, 29 (65.9%) were male patients, 26 (59.1%) patients had positive biopsy results, and the median follow-up period was 1643 days. Significantly more patients with alternate-day GC treatment used concomitant immunosuppressants (11 [84.6%] vs 11 [35.5%]; *P* = .007). The alternate-day strategy significantly lowered the GTI score after adjusting for cumulative GC dose until the assessment and PS (adjusted coefficient: −29.5 [−54.3, −4.8], *P* = .021 at 12 months; −20.0 [−39.8, −0.1], *P* = .049 at 24 months). Serious infections were numerically less frequent in the alternate-day group (incidence ratio [95% confidence interval [CI]: 0.45 [0.05, 3.63], *P* = .45). Most patients (92.3%) in the alternate-day GC treatment group and all patients in the daily GC treatment group showed treatment responses in the remission induction therapy. The PS-adjusted hazard ratio of alternate-day GC treatment for disease flares was not significant (1.55 [0.53, 4.51]; *P* = .43).

The alternate-day treatment strategy significantly reduced GC-related adverse events regardless of the cumulative GC dose. Alternate-day GC treatment is a feasible option for patients with IgG4-RD, without a significant increase in disease flares particularly when combined with immunosuppressants.

## 1. Introduction

Immunoglobulin G (IgG)4-related disease (IgG4-RD) is a fibroinflammatory disease affecting various organs^[1–7]^ and is rare with an estimated prevalence of 62 per million people.^[8]^ IgG4-RD is pathologically characterized by dense lymphoplasmacytic infiltrates enriched in IgG4-positive plasma cells and a variable degree of fibrosis that has a characteristic “storiform” pattern.^[9]^ In IgG4-RD, glucocorticoids (GCs) are the standard therapy,^[10]^ and patients often need to be maintained on long-term GC therapy because of frequent relapses during or after tapering of GC treatment.^[11]^ This causes concern about adverse effects caused by long-term GC use.

Alternate-day GC treatment is an effective strategy that can reduce adverse events in patients with rheumatoid arthritis^[12]^ and other autoimmune rheumatic diseases^[13]^ including polymyositis^[14]^ and giant cell arteritis.^[15]^ Alternate-day GC treatment strategy can be feasible in patients with IgG4-RD because they are often middle- or advanced-aged patients with comorbidities who need to avoid glucocorticoid-induced adverse events, and IgG4-RD has mild forms of the disease without severe organ damage, such as Mikulicz disease. However, there are no reports on the safety and effectiveness of the alternate-day GC strategy in patients with IgG4-RD.

Therefore, we conducted a retrospective cohort study to investigate the advantages and efficacy of alternate-day GC treatment in patients with IgG4-RD. Using the glucocorticoid toxicity index (GTI)^[16]^ and disease control, we compared GC-related adverse events between patients treated with alternate-day GC and those treated with daily GC.

## 2. Patients and Methods

### 2.1. Study participants

The medical records of patients with IgG4-RD who visited the Immuno-Rheumatology Center at St. Luke’s International Hospital, a tertiary center in Tokyo, Japan, between January 2004 and November 2020 were retrospectively reviewed. Patients who fulfilled the possible, probable, and definite comprehensive diagnostic criteria for IgG4-RD (2011)^[17]^ were included. Therefore, positive biopsy results (infiltration of IgG4 + plasma cells: ratio of IgG4+/IgG + cells >40% and >10 IgG4 + plasma cells/high-power field) and/or elevated IgG4 titer (≥135 mg/dL) were required for inclusion. Patients followed up for less than a year, those without pharmacological treatments, those initially treated at another hospital, and patients with overlapping autoimmune rheumatic or hematological diseases were excluded.

The patients were retrospectively assigned to two groups based on their treatment protocol. Patients who initiated treatment with the alternate-day or daily GC treatment strategies were included in the alternate-day or daily GC groups, respectively. Patients who switched from the daily GC regimen to the alternate-day protocol within 1 month after treatment initiation were included in the alternate-day group, even though they were initially treated with daily GC.

### 2.2. Data collection

Patients’ data were retrieved from the electronic medical records system. Data on the patients’ profiles, IgG4-RD status including IgG and IgG4 titers, disease flares, pathology results, organ involvement, and GC treatment strategy, as well as immunosuppressant (IS) use, were collected. Cumulative GC doses were calculated, and GC-related adverse events were identified by reviewing the electronic medical records. GC doses were presented as the daily prednisolone-equivalent dose (mg/day).

### 2.3. Safety assessment

#### 2.3.1. Evaluation of glucocorticoid-related and other adverse events.

Our primary outcome was the presence of a GC-related adverse event evaluated based on the GTI, which was assessed at 6, 12, 18, and 24 months. To decrease the influence of differences in daily GC doses and confounding in treatment indications, the effect of alternate-day GC treatment on the GTI was assessed using multivariate linear analysis adjusted for cumulative GC dose and propensity scores (PSs). The GTI is composed of nine domains, including body mass index, glucose tolerance, blood pressure, lipid metabolism, bone mineral density, myopathy, skin, neuropsychiatric toxicity, and infections. Scores are assigned to each domain, with the total score ranging from −36 to 439.^[16]^ Patients with higher scores had more GC-related damage.

Considering the adverse events caused by IS use, we also assessed adverse events including infections, leukocytopenia (<3000 × 10^3^/μL), anemia (hemoglobin ≤10 g/dL), thrombocytopenia (≤12.0 × 10^4^/μL), and liver dysfunction (aspartate aminotransferase ≥65 IU/L or alanine aminotransferase ≥75 IU/L, defined as a level more than twice as high as the reference range of our institutional laboratory). Serious infections were defined as infections requiring intravenous antibiotics or hospitalization or those resulting in death.

### 2.4. Efficacy assessment

#### 2.4.1. Igg4-related disease flares.

The efficacy of alternate-day GC treatment was assessed based on the occurrence of IgG4-RD flares. IgG4-RD flare was defined as a disease for which physicians intensified treatment (increased dose or initiated GC or IS) with clinical, radiological, or serological evidence of worsening disease. It was not considered a flare if the physician added or increased IS solely for further GC reduction without signs of a flare. The point and cumulative GC doses at months 6, 12, 18, and 24 were calculated. The GC dose at the time of the flare and the proportion of patients who discontinued GC were also evaluated.

### 2.5. Statistical analyses

Categorical variables were expressed as numbers (percentages) and quantitative variables as means (standard deviation) or medians (interquartile range [IQR]) with normal and non-normal distributions. In the univariate analysis, the chi-square or Fisher exact test was used to assess the categorical variables, and for continuous variables, the t-test or Wilcoxon rank-sum test was used appropriately based on their distributions.

The patients’ characteristics were summarized and compared between the alternate-day and daily GC treatment groups. To reduce confounding due to treatment indication of alternate-day and daily GC, the PS for the probability of adopting the alternate-day GC strategy was calculated using patient age, sex, IgG and IgG4 titers before treatment initiation, biopsy results, and the number of affected organs. For the assessment of adverse events, GTI scores and other events were compared at months 6, 12, 18, and 24 using univariate analysis. Moreover, univariate and multivariate linear regression analyses were performed to assess the effect of alternate-day GC treatment on GTI scores. In the multivariate analysis, the result was adjusted for cumulative GC dose until the assessment and PS. Missing values were imputed using the last observation carried forward method. We also analyzed the effect of alternate GC treatment on infections using a Poisson regression model.

For efficacy assessment, Kaplan–Meier curves and log-rank tests were used to evaluate the differences in flare-free survival between the groups. Cox multivariate regression analysis was used to evaluate the PS-adjusted hazard ratio of alternate-day GC treatment for flares.

For all analyses, a *P* value <.05 was considered significant. All analyses were performed using STATA software (version 16.1, StataCorp, College Station, TX, USA). The study was approved by the Institutional Review Board of St. Luke’s International Hospital (number: 20-R177) and was carried out according to the principles of the Helsinki Declaration. The need for informed consent was waived by the review board owing to the retrospective design of the study with provisions for opting out.

## 3. Results

### 3.1. Baseline characteristics

**Figure 1.**
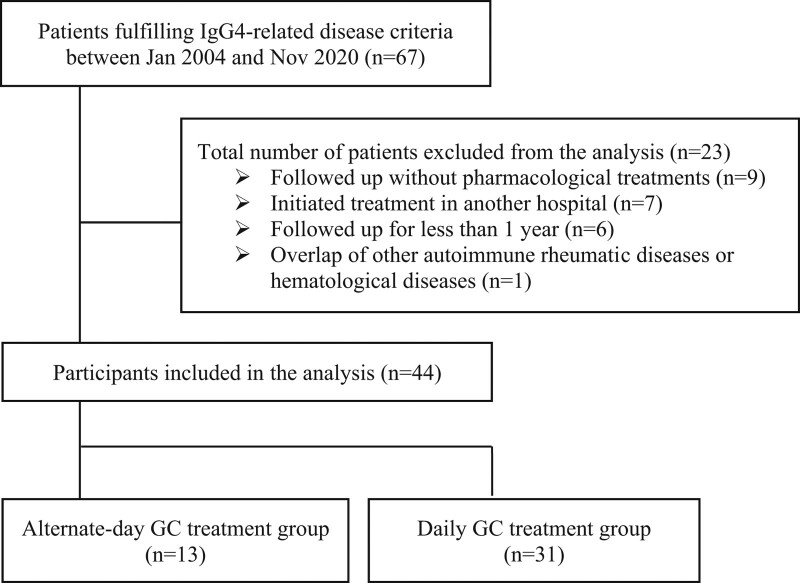
Flow chart of patient inclusion and exclusion. GC = glucocorticoid, IgG4 = immunoglobulin G4.

Among the 67 patients who fulfilled the possible, probable, and definite criteria of IgG4-RD, 23 patients, including a patient who exhibited symptoms that overlapped with Castleman disease, were excluded for the reasons shown in Figure 1. A total of 44 patients were included; 13 and 31 patients were classified into the alternate-day and daily GC treatment groups, respectively (Fig. 1). The patients’ baseline characteristics are summarized in Table 1. The median (IQR) age was 64 (60–70) years, 29 (65.9%) were male patients, 26 (59.1%) patients had positive biopsy results, and the median follow-up period was 1643 (871–2663) days. Lacrimal and salivary gland involvements were significantly more frequent in the alternate-day GC treatment group, and the number of involved organs also tended to be higher. Patients in the alternate-day group used concomitant IS before flare significantly more frequently than those in the daily GC group (11 [84.6%] vs 11 [35.5%]; *P* = .007). The calculated PS could estimate the use of an alternate GC treatment strategy with c-statistics of 0.82 [0.67, 0.97].

**Table 1:** Baseline characteristics.

	Overall (n = 44)	Alternate-day GC treatment (n = 13)	Daily GC treatment(n = 31)	*P* value
Age	64 [60, 70]	62 [54, 70]	65 [61, 70]	.67
Male (%)	29 (65.9)	6 (46.2)	23 (74.2)	.092
IgG4 high or biopsy-proven	44 (100.0)	13 (100.0)	31 (100.0)	–
High IgG4 (≥135 mg/dL) (%)	42 (95.5)	11 (84.6)	31 (100.0)	.082
Biopsy-proven (%)	26 (59.1)	10 (76.9)	16 (51.6)	.21
Follow-up (days)	1643 [871, 2663]	920 [751, 1883]	1696 [1027, 2799]	.19
Organ involvement				
Lacrimal (%)	16 (36.4)	8 (61.5)	8 (25.8)	.040
Orbital (%)	0 (0.0)	0 (0.0)	0 (0.0)	–
Salivary (%)	13 (29.5)	7 (53.8)	6 (19.4)	.033
Sinus (%)	4 (9.1)	2 (15.4)	2 (6.5)	.57
Thyroid (%)	2 (4.5)	1 (7.7)	1 (3.2)	.51
Lung (%)	7 (15.9)	2 (15.4)	5 (16.1)	>.99
Pancreas (%)	21 (47.7)	4 (30.8)	17 (54.8)	.19
Biliary tract (%)	1 (2.3)	0 (0.0)	1 (3.2)	>.99
Kidney (%)	4 (9.1)	0 (0.0)	4 (12.9)	.30
Retroperitoneal (%)	14 (31.8)	4 (30.8)	10 (32.3)	>.99
Prostatitis (%)	1 (2.3)	0 (0.0)	1 (3.2)	>.99
Periaortitis (%)	6 (13.6)	1 (7.7)	5 (16.1)	.65
Periarteritis (%)	4 (9.1)	2 (15.4)	2 (6.5)	.57
Joint (%)	0 (0.0)	0 (0.0)	0 (0.0)	–
Lymph node (%)	11 (25.0)	5 (38.5)	6 (19.4)	.26
Skin (%)	1 (2.3)	0 (0.0)	1 (3.2)	>.99
Number of involved organs	2.0 [1.0, 3.0]	3.0 [2.0, 3.0]	2.0 [1.0, 3.0]	.079
Multiple organ involvement (≥3 organs) (%)	20 (45.5)	8 (61.5)	12 (38.7)	.20
IS use before flare (%)	18 (41.0)	9 (69.2)	9 (29.0)	.013
IS use duringthefirst 12 mo (%)	19 (43.2)	10 (76.9)	9 (29.0)	.007
IS use duringthestudy period (%)	22 (50.0)	11 (84.6)	11 (35.5)	.007
Azathioprine (%)	3 (6.8)	1 (7.7)	2 (6.5)	>.99
Methotrexate (%)	12 (27.3)	6 (46.2)	6 (19.4)	.14
Mizoribine (%)	20 (45.5)	10 (76.9)	10 (32.3)	.009
Mycophenolate Mofetil (%)	0 (0.0)	0 (0.0)	0 (0.0)	–
Tacrolimus (%)	2 (4.5)	1 (7.7)	1 (3.2)	.51

Continuous variables are presented as mean (standard deviation) or median [interquartile range].

GC = glucocorticoid, IgG4 = immunoglobulin G4, IS = immunosuppressants.

### 3.2. Safety assessment

#### 3.2.1. Glucocorticoid dose.

The GC doses are summarized in Table 2. The initial GC dose was significantly lower in the alternate-day GC treatment group (17.5 [15–30] vs 30 [30–40]; *P* = .002). The cumulative GC doses between 0 and 6 months, 6 and 12 months, and 12 and 18 months were also significantly lower in the alternate-day GC treatment group (1781 [1525–3058] vs 2975 [2475–3541], *P* = .017; 760 [458–1053] vs 1082.25 [915–1483], *P* = .034; and 459 [296–816] vs 910 [660–990], *P* = .034, respectively).

**Table 2:** Single time point and cumulative glucocorticoid doses and glucocorticoid discontinuation.

	Alternative day GC treatment (n = 13)	Daily GC treatment (n = 31)	*P* value
GC dose (mg/day)			
At treatment initiation	17.5 [15, 30]	30 [30, 40]	.002
At 6 mo	5.0 [5, 7.5]	7.5 [5, 10]	.30
At 12 mo	3.0 [2, 5]	5.0 [4.5, 7.5]	.008
At 18 mo	2.5 [1, 5]	5.0 [2.5, 5]	.15
At 24 mo	2.5 [1, 5]	4.0 [2, 5]	.25
At latest visit	2.5 [1, 2.5]	2.5 [1, 5]	.26
Cumulative GC dose (mg)			
Between 0 and 6 months	1781 [1525, 3058]	2975 [2475, 3541]	.017
Between 6 and 12 months	760 [458, 1053]	1082 [915, 1483]	.034
Between 12 and 18 months	459 [296, 816]	910 [660, 990]	.034
Between 18 and 24 months	305 [45, 659]	809 [458, 915]	.069
After 24 mo (per half year)	710 [548, 977]	1027 [727, 1824]	.086
GC discontinuation	6 (46%)	10 (32%)	.50
Successful disease control without GC for more than 1 year	5 (38%)	8 (26%)	.48
GC dose at flare (mg/day)	1.6 [0, 5]	2.5 [0, 5]	.55

Data are presented as median [interquartile range] for continuous variables and n (%) for categorical variables.

GC = glucocorticoid.

#### 3.2.2. Glucocorticoid toxicity index and other adverse events.

The GTI score was significantly lower in the alternate-day GC treatment group at 12, 18, and 24 months, as shown in Table 3. Patients treated with the alternate-day GC strategy tended to have fewer adverse events in each domain of the GTI globally; however, there were no significant differences (see Table S1, http://links.lww.com/MD/H458, Supplemental Digital Content, http://links.lww.com/MD/H458, which summarizes the factors of the GTI). None of the patients in either group exhibited GC-associated myopathy.

**Table 3:** Summary of the glucocorticoid toxicity index and other adverse events.

	Alternate-day GC treatment (n = 13)	Daily GC treatment (n = 31)	*P* value
GTI			
At mo 6	10 [0, 19]	19 [0, 59]	.23
At mo 12	0 [−9, 0]	19 [0, 51]	.005
At mo 18	0 [0, 10]	19 [2, 40]	.012
At mo 24	0 [−10, 10]	19 [11, 47]	.001
Infections			
Serious bacterial infection	1 (8%)	6 (19%)	.65
Serious bacterial infection rate	1.81 (0.04, 10.1)	4.06 (1.6, 8.4)	.45
Herpes zoster	0 (0%)	4 (13%)	.30
Herpes zoster rate	0 (0, 6.7)	2.32 (0.6, 5.9)	.99
Leukocytopenia	1 (8%)	1 (3%)	.48
Anemia	2 (17%)	6 (19%)	>.99
Thrombocytopenia	1 (8%)	4 (12%)	>.99
Liver dysfunction	2 (17%)	7 (22%)	>.99

Data are presented as median [interquartile range] for continuous variables and n (%) for categorical variables. The incidence rate is presented as per 100 person-years (95% confidence interval).

GC = glucocorticoid, GTI = glucocorticoid toxicity index.

**Figure 2.**
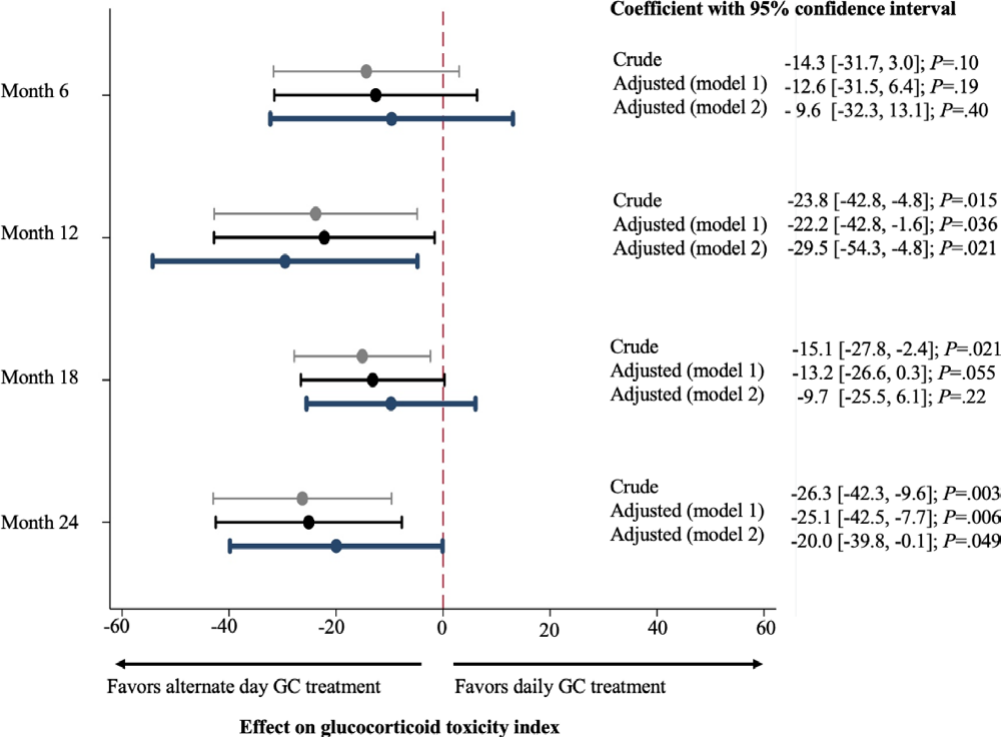
Crude and adjusted coefficients of alternate-day glucocorticoid strategy for glucocorticoid toxicity index in univariate and multivariate linear regression analysis. Gray and black lines indicate crude and adjusted coefficients with 95% confidence intervals. In the multivariate model, the effect of alternate-day glucocorticoid treatment was adjusted for cumulative GC dose until the assessment and propensity score. GC = glucocorticoid.

Crude and adjusted β coefficients of alternate-day GC treatment for GTI scores in a linear regression model are shown in Figure 2. The mean β coefficients were consistently negative for each assessment, indicating that the alternate-day GC was protective against GC-related adverse events. After adjusting for cumulative GC dose until each assessment, the alternate-day GC strategy was a significant protective factor for GC-related toxicity at 12 and 24 months (β coefficient with 95% confidence interval [95% CI]: −22.2 [−42.8, −1.6]; *P* = .036 and −25.1 [−42.5, −7.7]; *P* = .006, respectively). The result was preserved when the result was adjusted for cumulative GC dose and PS (β coefficient [95% CI]: −29.5 [−54.3, −4.8]; *P* = .021 at 12 months and −20.0 [−39.8, −0.1]; *P* = .049 at 24 months).

There were no significant differences in other adverse events (Table 2). Serious infections were numerically lower in the alternate-day GC group, but the difference was not statistically significant. The incidence rate ratio (95% CI) of serious infections in the alternate-day GC treatment group was 0.45 ([0.05, 3.63], *P* = .45). None of the patients in the alternate-day GC group had herpes zoster during the study period.

### 3.3. Efficacy assessment

#### 3.3.1. Treatment responses and igg4-related disease flares.

**Figure 3.**
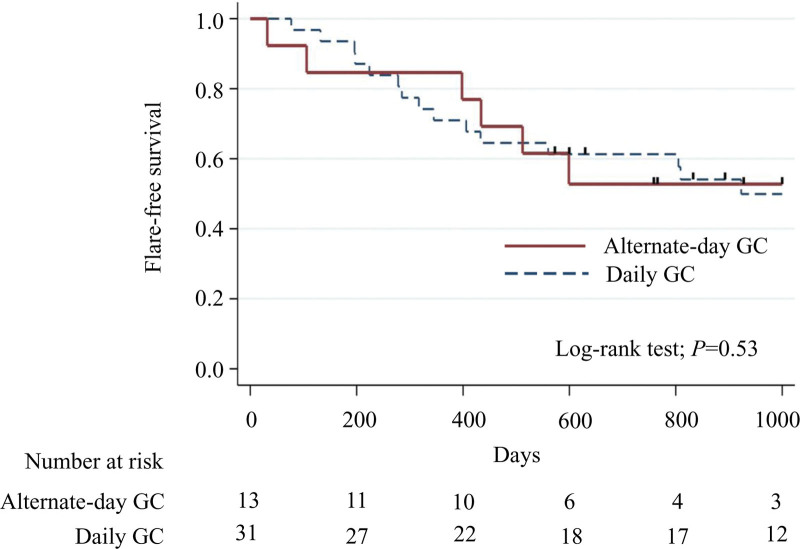
Flare-free survival of patients with alternate-day and daily glucocorticoid treatment strategies. GC = glucocorticoid.

Most patients (92.3%) in the alternate-day GC treatment group and all patients in the daily GC treatment group showed treatment responses in remission induction therapy. Kaplan–Meier curves for disease flare-free survival according to the GC treatment strategy are shown in Figure 3. There were no significant differences in IgG4-RD flare between the alternate-day and daily GC treatment groups based on the log-rank test (*P* = .53). The Cox proportional hazard model showed that alternate-day GC treatment was not a significant risk factor for IgG4-RD flares (1.55 [0.53, 4.51]; *P* = .43) after adjusting for PS. The result was preserved even after adjusting for IS use before flares. There were no significant differences in the proportion of patients who discontinued GC or were controlled without GC for more than 1 year (6 [46%] vs 10 [32%]; *P* = .50 and 5 [38%] vs 8 [26%]; *P* = .48, respectively). Moreover, the median (IQR) GC dose (mg/day) at the time of disease flare was 1.6 (0–5) in the alternate-day GC treatment group and 2.5 (0–5) in the daily GC treatment group (*P* = .55) (Table 2).

## 4. Discussion

This study revealed that an alternate-day GC treatment strategy can contribute to lower GC-related toxicity without significantly increasing the risk of relapse, particularly when combined with ISs. To the best of our knowledge, this is the first report to demonstrate the advantages of an alternate-day GC treatment strategy in patients with IgG4-RD. While cumulative GC doses tended to be lower in the alternate-day GC treatment group, the alternate-day GC strategy exhibited lower rates of GC-related toxicity even after adjusting for cumulative GC doses and PS. The alternate-day GC strategy can be considered a viable treatment option and particularly suitable for patients who are vulnerable to GC toxicity, such as older patients with comorbidities, such as hypertension, dyslipidemia, and diabetes mellitus.

Similar to our study, a previous report on alternate GC regimens in polymyositis showed fewer adverse events compared to the daily dose regimen.^[14]^ Worsening of hypertension also tended to be less frequent in the alternate GC treatment group in the current study, as previously reported.^[18]^ It is reasonable that the current study showed worsening of diabetes less frequently in the alternate-day GC group considering that alternate-day GCs are associated only with alternate-day hyperglycemia.^[19]^ Moreover, serious infection and herpes zoster rates were numerically lower in the alternate-day GC treatment group despite the more frequent use of IS. This result is in agreement with those of previous studies,^[12,14]^ possibly because the leukocyte function is maintained in patients treated with an alternate-day GC strategy.^[20]^ Because many previous studies lacked concrete measures to determine GC-related toxicity, greater clinical use of the GTI is required to evaluate the effects of alternate-day GC treatment. In this study, significant differences were revealed by using the GTI, although there were no significant differences in any of the domains observed in this study, possibly due to the small number of patients.

Additionally, there were no significant differences in other adverse events, such as cytopenia or liver dysfunction between the groups, despite the frequent use of IS in the alternate-day GC group. These results suggest an advantage in the safety of alternate-day GC treatment, even when combined with IS.

Regarding the control of IgG4-RD, our study showed that the risk of flares did not significantly increase with alternate-day GC treatment after PS adjustment. However, it should be noted that patients in the alternate-day GC treatment group had more frequent use of ISs which are effective for controlling IgG4-RD.^[21–24]^ The effectiveness of the alternate-day GC regimen has been controversial. Some previous studies have shown insufficient control of diseases while others have not.^[15,25]^ However, there are various clinical phenotypes^[26,27]^ in IgG4-RD, including mild forms of the disease without severe organ damage. The safety of the treatment should be prioritized, particularly in these mild cases of IgG4-RD.

The strength of this study is the inclusion of detailed data regarding GC doses and GC-associated toxicity using the GTI. Participants were followed for a long period, with a median time of 1643 days. Moreover, we calculated the PS and adjusted the cumulative GC dose to reduce confounding effects when evaluating the GTI. Our study has some limitations. First, this was a preliminary single-center retrospective study with a relatively small cohort, while IgG4-RD is a rare disease. Second, there were differences in patient baseline characteristics between the treatment groups. Patients with alternate-day GC tend to have less severe manifestations. However, the result was adjusted by PS to reduce the confounding factors according to the indications. Finally, there was significantly greater use of ISs in the alternate-day GC treatment group. However, it can be concluded that alternate-day GCs contributed to lowering the GC-related toxicity without increasing flares, at least in combination with ISs.

In conclusion, this study revealed the advantages of an alternate-day GC strategy in decreasing GC-related toxicity without increasing IgG4-RD flare-ups, particularly when combined with ISs. Alternate-day GC strategies should be considered as treatment options for IgG4-RD, particularly in patients who are vulnerable to GC toxicity. Prospective studies with larger cohorts are required to confirm the findings of this study.

## Acknowledgments

We appreciate the contributions of all physicians, nurses, and other staff who cared for the enrolled patients. We wish to thank Ms. Horikawa, Mr. Nakashima, and the Information System Unit at St. Luke’s International Hospital for their support with data collection.

## Author contributions

SF contributed to the original conception and design of this study, which TN, SK, YI, MS, AN, HT, MK, SO, and MO reviewed and corrected. SF, TN, and SK collected data. SF performed data analysis with support from SO. SF interpreted the data, and YI, MS, AN, HT, MK, SO, and MO advised on and modified the interpretation. SF drafted the original manuscript, which was critically reviewed and revised by all other authors (TN, SK, YI, MS, AN, HT, MK, SO, and MO). All authors have read and approved the final version of the manuscript.

**Conceptualization:** Sho Fukui, Hiromichi Tamaki, Masato Okada.

**Data curation:** Sho Fukui, Takehiro Nakai, Satoshi Kawaai.

**Formal analysis:** Sho Fukui, Sachiko Ohde.

**Investigation:** Sho Fukui.

**Methodology:** Sho Fukui, Sachiko Ohde.

**Project administration:** Sho Fukui, Masei Suda, Masato Okada.

**Supervision:** Takehiro Nakai, Satoshi Kawaai, Yukihiko Ikeda, Masei Suda, Atsushi Nomura, Hiromichi Tamaki, Mitsumasa Kishimoto, Masato Okada.

**Writing – original draft:** Sho Fukui.

**Writing – review & editing:** Takehiro Nakai, Satoshi Kawaai, Yukihiko Ikeda, Masei Suda, Atsushi Nomura, Hiromichi Tamaki, Mitsumasa Kishimoto, Sachiko Ohde, Masato Okada.

## Supplementary Material


