# Supplementary material for: Advantages of an alternate-day glucocorticoid treatment strategy for the treatment of IgG4-related disease: A preliminary retrospective cohort study
Source: Medicine (Baltimore). 2022 Sep 30;101(39):e30932. doi: 10.1097/MD.0000000000030932 (PMC9524883; doi:10.1097/MD.0000000000030932)
Supplement: Supplementary file 1 [file medi-101-e30932-s001.pdf]

Supplementary Table 1

Summary of each factor of the glucocorticoid toxicity index.

		Alternate-day GC treatment (n=13)	Daily GC treatment (n=31)	*P*-value
Body mass index				
at month 6	Improvement	0 (0%)	1 (3%)	>.99
	Moderate increase	0 (0%)	1 (3%)	
at month 12	Improvement	0 (0%)	1 (3%)	>.99
	Moderate increase	0 (0%)	1 (3%)	
at month 18	Improvement	0 (0%)	1 (3%)	>.99
	Moderate increase	0 (0%)	1 (3%)	
at month 24	Improvement	0 (0%)	2 (6%)	>.99
	Moderate increase	0 (0%)	1 (3%)	
Diabetes mellitus				
Medication use at baseline		2 (15%)	6 (19%)	>.99
at month 6	Improvement	0 (0%)	2 (6%)	.22
	Worsening	2 (15%)	3 (10%)	
	Worsening despite increased treatment	0 (0%)	7 (23%)	
at month 12	Improvement	0 (0%)	1 (3%)	.33
	Worsening	1 (8%)	9 (29%)	
	Worsening despite increased treatment	0 (0%)	3 (10%)	
at month 18	Improvement	1 (8%)	2 (6%)	.53
	Worsening	1 (8%)	8 (26%)	
	Worsening despite increased treatment	0 (0%)	3 (10%)	
at month 24	Improvement	1 (8%)	2 (6%)	.13
	Worsening	0 (0%)	7 (23%)	
	Worsening despite increased treatment	0 (0%)	2 (6%)	
Blood pressure				
Medication use at baseline		6 (46%)	11 (35%)	.49
at month 6	Improvement	3 (23%)	6 (19%)	.89
	Worsening	2 (15%)	8 (26%)	
at month 12	Improvement	3 (23%)	4 (13%)	.18
	Worsening	1 (8%)	11 (35%)	
at month 18	Improvement	3 (23%)	4 (13%)	.60
	Worsening	2 (15%)	8 (26%)	
at month 24	Improvement	3 (23%)	3 (10%)	.22
	Worsening	2 (15%)	14 (45%)	
	Worsening despite increased treatment	0 (0%)	2 (6%)	
Dyslipidemia				
Medication use at baseline		2 (15%)	5 (16%)	>.99
at month 6	Improvement	0 (0%)	3 (10%)	.64
	Worsening	4 (31%)	9 (29%)	

at month 12	Improvement	1 (8%)	3 (10%)	>.99
	Worsening	2 (15%)	4 (13%)	
at month 18	Improvement	0 (0%)	3 (10%)	.49
	Worsening	4 (31%)	4 (13%)	
	Worsening despite increased treatment	0 (0%)	1 (3%)	
at month 24	Improvement	1 (8%)	2 (6%)	>.99
	Worsening	2 (15%)	5 (16%)	
	Worsening despite increased treatment	0 (0%)	1 (3%)	
Bone mineral density				
at month 6	Worsening	0 (0%)	1 (3%)	>.99
at month 12	Worsening	0 (0%)	1 (3%)	>.99
at month 18	Worsening	0 (0%)	1 (3%)	>.99
at month 24	Worsening	0 (0%)	1 (3%)	>.99
Skin				
at month 6	Mild symptoms	0 (0%)	1 (3%)	>.99
at month 12	Mild symptoms	0 (0%)	1 (3%)	>.99
at month 18	Mild symptoms	0 (0%)	1 (3%)	>.99
at month 24	Mild symptoms	0 (0%)	2 (6%)	>.99
Neuropsychiatric symptom				
Medication use at baseline		0 (0%)	4 (13%)	.30
at month 6	Mild symptoms	1 (8%)	8 (26%)	.24
at month 12	Mild symptoms	0 (0%)	7 (23%)	.086
at month 18	Mild symptoms	1 (8%)	6 (19%)	.41
at month 24	Mild symptoms	0 (0%)	7 (23%)	.16
Myopathy	No myopathies during the study period	-	-	-
Infections				
~ month 6	Specific infections	0 (0%)	3 (10%)	.54
~ month 12	Infections ≥ grade 3	0 (0%)	1 (3%)	>.99
~ month 18	No specific or ≥ grade 3 infections	-	-	-
~ month 24	No specific or ≥ grade 3 infections	-	-	-

Data are presented as n (%) for categorical variables.

GC = glucocorticoid.
